# Friend or Foe: Lipid Droplets as Organelles for Protein and Lipid Storage in Cellular Stress Response, Aging and Disease

**DOI:** 10.3390/molecules25215053

**Published:** 2020-10-30

**Authors:** Florian Geltinger, Lukas Schartel, Markus Wiederstein, Julia Tevini, Elmar Aigner, Thomas K. Felder, Mark Rinnerthaler

**Affiliations:** 1 Department of Biosciences, University of Salzburg, 5020 Salzburg, Austria; geltingerfl@stud.sbg.ac.at (F.G.); lukas.schartel@stud.sbg.ac.at (L.S.); markus.wiederstein@sbg.ac.at (M.W.); 2 Department of Laboratory Medicine, Paracelsus Medical University, 5020 Salzburg, Austria; j.tevini@salk.at; 3 First Department of Medicine, Paracelsus Medical University, 5020 Salzburg, Austria; e.aigner@salk.at

**Keywords:** Lipid droplets, aging, proteostasis, mitochondria, lipotoxicity, neurodegenerative diseases, interorganelle communication

## Abstract

Lipid droplets (LDs) were considered as a mere lipid storage organelle for a long time. Recent evidence suggests that LDs are in fact distinct and dynamic organelles with a specialized proteome and functions in many cellular roles. As such, LDs contribute to cellular signaling, protein and lipid homeostasis, metabolic diseases and inflammation. In line with the multitude of functions, LDs interact with many cellular organelles including mitochondria, peroxisomes, lysosomes, the endoplasmic reticulum and the nucleus. LDs are highly mobile and dynamic organelles and impaired motility disrupts the interaction with other organelles. The reduction of interorganelle contacts results in a multitude of pathophysiologies and frequently in neurodegenerative diseases. Contacts not only supply lipids for β-oxidation in mitochondria and peroxisomes, but also may include the transfer of toxic lipids as well as misfolded and harmful proteins to LDs. Furthermore, LDs assist in the removal of protein aggregates when severe proteotoxic stress overwhelms the proteasomal system. During imbalance of cellular lipid homeostasis, LDs also support cellular detoxification. Fine-tuning of LD function is of crucial importance and many diseases are associated with dysfunctional LDs. We summarize the current understanding of LDs and their interactions with organelles, providing a storage site for harmful proteins and lipids during cellular stress, aging inflammation and various disease states.

## 1. Introduction

LDs have been established as genuine organelles with a relevant role in the control of cellular stress and various diseases. In the past, LDs were considered to be inert „oil bodies“ inside a cell, but have gained attention as organelles with a distinct proteome [1]. LDs originate from the endoplasmic reticulum (ER) and consist of a neutral lipid core, which is surrounded by a phospholipid monolayer [2]. Phosphatidylcholines predominate the monolayer [3], whereas the hydrophobic core consists of triacylglycerols (TAG), sterol esters [4], diacylglycerols (DAG), and monoacylether-DAG [5]. Numerous proteins including perilipins (PLIN; LD associated coating proteins) or DGAT1/2 (diacylglycerol acyltransferase) are integrated into or attached to the monolayer [5]. LDs are also storage organelles for lipids and contribute to energy homeostasis [6]. Energy storing LDs serve as vehicles for the maintenance of essential metabolic functions inside the cell during times of low energy availability [7]. LDs also physically attach to and connect different organelles including mitochondria, peroxisomes, the nucleus, ER, vacuoles and lysosomes [1]. Shuttled to mitochondria or peroxisomes, LDs directly donate fatty acids (FAs) for beta oxidation in mammals [8]. LDs are not only involved in the utilization and exchange of lipids but also contribute to cellular protein transport and distribution [9]. 

## 2. Special Features of LDs

The involvement of this organelle in diverse cellular processes is based on two properties: (1) LDs are “interactive” and can get in contact with most other organelles in the cell; (2) LDs can act as a “sink”, implying the function as a transient storage site for harmful proteins and lipids. Both features give LDs a unique role in the cell and help to explain why LDs are at the heart of various diseases.

### 2.1. LD Motility

The high motility of LDs contributes to and enables contact with a variety of other cell organelles. A recent study correlates LD velocity along a network of microtubules with tumor aggressiveness [10]. Increased nutrient requirements and rates of cargo protein transport in cancer cells may result in elevated LD velocity. LDs reach mean velocities around 1.45 µm/s up to 3.4 µm/s in aggressive PC-3 cancer cells. LD velocity can dose-dependently decrease after nocodazole treatment, an antineoplastic agent that negatively affects microtubule polymerization. In comparison to the trafficking speed of other organelles (peroxisomes 0.4 µm/s in *Yarrowia lipolytica* [11], lysosomes 0.2 µm/s in monkey kidney epithelial cells [12]), LDs are among the fastest moving cellular compartments. While organelle velocity measurements are not comparable *per se* due to different model systems or treatments, they still illustrate that LDs are highly mobile and dynamic organelles.

LDs can slowly move by random free diffusion and limited Brownian motion. For high motility, LDs utilize the cytoskeleton and molecular motor proteins such as kinesin, dynein and myosin [13]. These movements on actin or microtubules are uni- or bidirectional, and ethanol-induced hyperacetylation of microtubules leads to LD immobility in a hepatoma derived cell line [14]. Actin dependent LD transport occurs in *Drosophila melanogaster* cells via Myo1, and in *Arabidopsis thaliana* via myosin XIs [15,16]. In *Saccharomyces cerevisiae,* Myo2 mediates the active redistribution of LDs along microtubules to daughter cells during cell division [17].

*Schizosaccharomyes pombe* are yeast cells, which form spores that can persist for years. Lack of nitrogen induces the redistribution of LDs to spores in a process that depends on actin-polymerization [18]. Cells devoid of TAG synthesizing enzymes fail to relocate LDs and produce spores that are incapable to germinate. The observation highlights the importance of active LD distribution. Other proteins such as VPS13A modulate LD motility in the human fibroblast MRC5 cell line and *Drosophila* cells. VPS13A negatively regulates LD motility acting as a tether that keeps the ER and LDs in close proximity. This is most probably a prerequisite for the transfer of lipids from the ER to LDs. Consequently, VPS13A knockout cells have less LD/ER contact sites and increased motility of LDs [19]. Additionally it has been shown that LDs are able to “hitchhike” on early endosomes and move along microtubules in the fungal model system *Ustilago maydis* [20]. Generally it can be stated that impaired LD motility also affects the contact to other organelles with an impact on several human diseases as shown in the following section.

### 2.2. LD-Organelle Interactions

**Figure 1:**
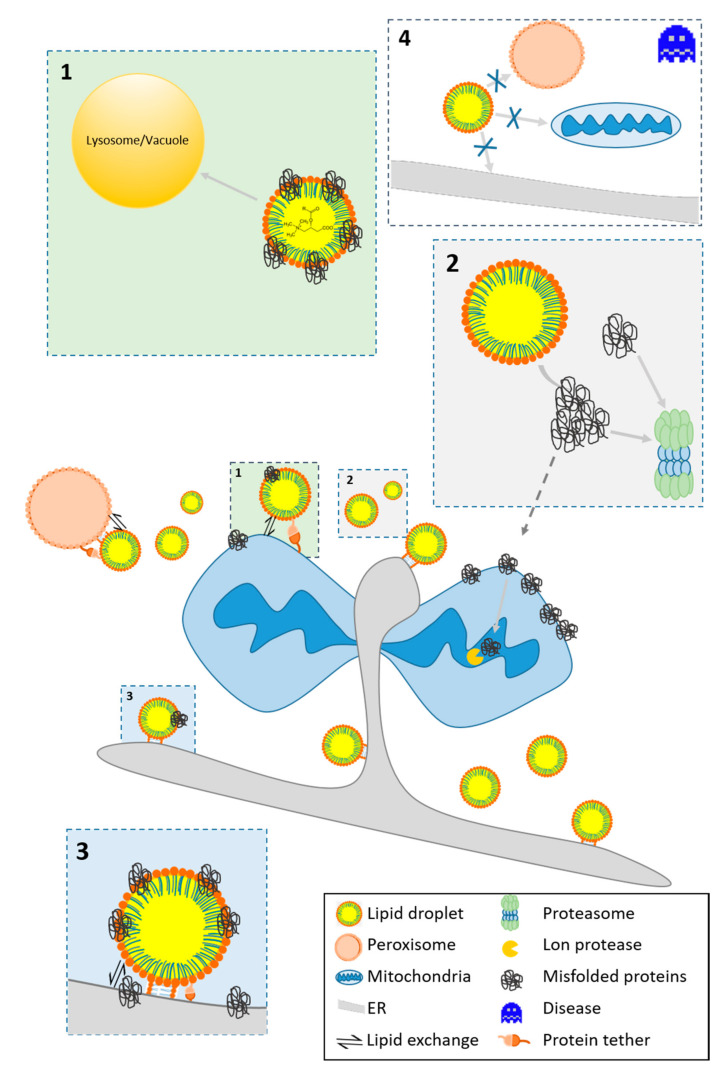
LDs as a storage organelle. In a trimeric complex LDs show a strong association with the ER and mitochondria. LDs constantly bud off the ER (birth of LDs) and are degraded in the vacuole/lysosome in an autophagic process called lipophagy (death of LDs) (**1**). During its life, LDs fulfil a multitude of functions. Among them is the role of LDs in protein homeostasis. LDs assist the 26S proteasome in dissolving protein aggregates by sequestering sterol derivatives (**2**). Stabilized by a protein tether and a lipidic bridge LDs interact with the ER and take up misfolded proteins after ERAD malfunction. Additionally, toxic lipids (e.g., lipid peroxides, acylceramides) accumulate in LDs (**3**). Similarly, harmful mitochondrial proteins can be relocalized from the OMM to LDs. Finally, these loaded LDs degrade in the lysosome/vacuole (**3**). A failure in interorganelle contacts results in a multitude of disease (especially neurodegenerative diseases) (**4**).

The scope of LD interactions with other organelles is broad and some excellent reviews already cover this topic in depth [6,21]. We therefore only give a summary of LD interactions and focus on some of the involved key mechanisms and proteins. Membrane contact sites form between several organelles and are not specific for LDs (Figure 1). 

Such interactions are essential to exchange signals, membrane lipids and proteins. One example for inter-organelle communication is via vesicles (e.g., COPII (coat protein complex II) vesicles that bud off from the ER and fuse with the Golgi apparatus) [22]. Another example is the close apposition (less than 80 nm) between membranes of two organelles and their stabilization by proteins. These contact sites can form between two identical or between different organelles [23]. More direct in its nature is the mitochondrial “kiss-and-run” mechanism in which two mitochondria transiently fuse to exchange lipids and proteins [24].

The formation of “lipidic bridges” is LD specific and occurs between the ER and LDs as observed by electron microscopy. Such bridges form a continuum between the phospholipid monolayer of LDs and the outer leaflet of the ER bilayer [8]. Importantly, “lipidic bridges” do not cause the inter-mixing of membranes, probably due to the high surface tension of LDs. The high surface tension of LDs originates in their unique lipid composition, where a dynamic phospholipid monolayer encloses neutral lipids such as TAGs and sterol esters. The lipid composition of the monolayer as well as the size of LDs modulate surface tension. Smaller-LDs have a significantly higher surface tension than larger ones [25]. LD specific proteins diffuse from the ER to LDs after their insertion into the ER bilayer. Lipidic bridges can also connect LDs and peroxisomes [26], and may be necessary for all LD-organelle interactions. The rigid bridges can persist for a long time, as in yeast cells where LDs mainly remain in close association with the ER [27]). To establish the required rigidness, protein tethers additionally stabilize the LD contact sites.

LDs originate from the ER, and various proteins can re-establish LD-ER contacts even after LD “bud off”. Such connectors are DGAT2 or FATP1, a FA transport protein with acyl-CoA synthetase activity. Both proteins promote LD growth by synthesizing and transferring TAG to LDs [28]. The Rab18-NRZ-SNARE complex is another connecting structure in preadipocytes [29]. Not only multi-protein complexes, but also some monomeric structures may act as a clamp between the ER and LDs. One ER-LD tether is sortin nexin 14 (SNX14), an ER associated protein that binds LDs via its transmembrane domains while still anchored to the ER bilayer [30]. Mutations in SNX14 lead to a neuronal disease known as Distinctive Autosomal-Recessive Cerebellar Ataxia (see Section 5) [31]. Another monomeric link is the ER and LD resident protein VPS13A [19], which may also localize to mitochondria. Consequently, deletion of VPS13A causes decreased ER-mitochondrial contacts. VPS13A contributes to many diverse interorganelle membrane contact sites [19]. Similar to mutations in genes that encode proteins involved in the maintenance of organelle tethers, mutations in VPS13A result in neuronal disorders. One example is autosomal recessive Chorea-acanthocytosis [32], which will be described later.

PLINs are members of a protein family that associate with the surface of LDs, and PLIN5 is of high importance for the interaction of LDs with mitochondria. PLIN5 is highly dynamic and attaches to LDs in dependence on the physiological state of the cell (see Section 3.2.2 and Section 4.2). Emphasizing the importance of LD junction deficiencies, PLIN5 dysfunction causes diseases such as neutral lipid storage disease (Section 5.2) and hepatocellular carcinoma (HCC) (Section 5.4). 

Additional molecular connections occur in different cell types. PLIN1 interacts with mitofusin 2 (MFN2) in the outer mitochondrial membrane (OMM) [33], and is necessary to maintain transfer of FAs to mitochondria in brown adipose tissue. MFN2 associates with many common neuropathies including Parkinson’s disease [34,35].

Mitochondria in white adipose tissue contain mitoguardin 2 in the OMM, which also contributes to mitochondrial fusion. Mitoguardin 2 contains amphipathic segments that can target LDs and acts as a monomeric LD-mitochondrial tether [36]. Additional candidates involved in the formation of LD-mitochondrial connections were identified in yeast cells. LD proteins Erg6 and Pet10 physically interact with mitochondrial Mcr1. Pet10 is homologous to mammalian PLIN1, whereas Erg6 and Mcr1 are both involved in ergosterol biosynthesis, the yeast main sterol [37].

Connections between LDs and peroxisomes have long been observed, but the underlying mechanisms are unidentified [26]. Peroxisomes possess the ability to oxidize very long chain FAs through β-oxidation and branched-chain phytanic acid by α-oxidation. Only recently the M1 isoform of the ATPase spastin, was identified as a protein that facilitates LD-peroxisome contact [38]. Spastin localizes to LDs via an N-terminal hydrophobic hairpin motif and interacts with the long chain FA transporter ABCD1 via a peroxisome interacting region motif. Both proteins can contribute to the development of diseases, namely hereditary spastic paraplegia and X-linked adrenoleukodystrophy as will be discussed in the neurodegenerative disease section. 

Of all interactions between LDs and organelles, the contact to the vacuole/lysosome (vacuole is the yeast lysosome) is least understood. LDs are internalized into the vacuole/lysosome in a process called lipophagy, the autophagic process for the mobilization of free FAs, lipid homeostasis and LD turnover. In yeast cells, LDs directly interact with the vacuolar membrane and stimulate its invagination. This strictly growth-phase dependent process was termed microlipophagy [39]. Protein tethers are not involved in this process, but LDs interact with sterol enriched microdomains in the vacuolar membrane [40]. Nonetheless, other proteins such as sterol transporters Ncr1 and Ncr2, MDM1 and autophagy-specific Atg14 modulate the LD invagination process [41,42]. Mdm1 is part of nucleus-vacuole junctions, a complex that tethers the ER to vacuoles. Mdm1 also associates with LDs, indicating a trimer organelle interaction (ER-LD-vacuole). In fact, a close association of LDs with the nucleus-vacuole junctions was observed [21,43].

Mammals have two alternative autophagic programs, termed macrolipophagy and chaperone-mediated autophagy. During macrolipophagy, a double-layered membrane coats LDs [44]. The resulting autophagosome is transported to and fuses with the lysosome before LDs are released. In addition to core autophagic components, some members of the Rab GTPase family (RAB7 and RAB10) on the LD surface are important for the process [45]. Of special interest is the protein huntingtin that modulates autophagy and upon deletion abolishes macrolipophagy [46]. In fact, mutations in huntingtin cause the accumulation of LDs and influences the development of Huntington’s disease (see Section 5.1).

The heat shock cognate 71 kDa protein (HSC70) is essential for chaperone mediated autophagy. HSC70 recognizes proteins that contain the pentapeptide motif KFERQ and mediates their degradation in the lysosome. PLIN2 and PLIN3 harbour such a motif and are substrates in this autophagic process. Degradation of PLIN2 and PLIN3 is a prerequisite for subsequent lipolysis [47]. These observations may support the general view that malfunctions in the establishment of protein tethers and “lipid bridges” between LDs and a broad variety of organelles contribute to several disease states, especially neuronal diseases. 

LDs are actively involved in the cellular network and get in contact with diverse organelles. Both processes, the formation of contacts with other membranes as well as the directed transport of proteins onto LDs, require exquisite fine-tuning. During imbalance of cellular lipid homeostasis, LDs can also support cellular detoxification, as shown in the following section.

## 3. The Role of LDs in Cellular Detoxification

### 3.1. Lipotoxicity

Lipotoxicity displays a state of lipid dyshomeostasis, where the uptake, storage and distribution of lipids is impaired [48]. It results from the accumulation of specific lipid intermediates in several tissues, which cause cellular dysfunction and death. LDs can serve as reservoir for essential FAs, and as a deposit for damaged FAs. The LD monolayer mainly consists of phosphatidylcholine (PC), phosphatidylethanolamine (PE) and phosphatidylinositol (PI). Saturated and unsaturated FAs are present in these phospholipids, and the latter class includes monounsaturated (MUFAs) and polyunsaturated FAs (PUFAs). The composition of the LD monolayer, especially the distribution of MUFAs and PUFAs within this monolayer influences its fluidity as well as biophysical properties [49]. PUFAs within the monolayer also serve as precursors of lipid mediators such as eicosanoids [50], which modulate immune and inflammatory responses and their resolution. Furthermore, LDs provide an active site for eicosanoid synthesis out of arachidonyl lipids [50]. With losing their role as regulatory hubs in lipid homeostasis, they can also contribute to lipotoxcity. 

Free FAs are generally toxic within cells, as they act like detergents and may disrupt membranes and proteins. Especially free saturated palmitate (C16:0) can have cytotoxic effects, which contribute to the development of atherosclerosis, type 2 diabetes as well as diabetic cardiomyopathy [51,52]. Here LDs can act as reservoir for palmitate to mitigate its lipotoxcic effects [53].

In contrast, some MUFAs seem less toxic or even are cytoprotective. Recently, the role of the MUFA oleate (C18:1) has been discussed controversially in literature. Some studies report oleate as a toxic FFA [54], whereas others report that oleate can attenuate palmitate induced apoptosis. Free palmitate can be used for TAG biosynthesis. In this way LDs act as a buffer for palmitate and due to its uptake into LDs lipotoxicity is reduced [9,48]. Metabolic intermediates of TAG metabolism, such as acyl-coenzyme A, lysophosphatidic acid (LPA), phosphatidic acid (PA) as well as di- and monoacylglycerol also contribute to lipotoxicity. These metabolites are signalling lipids and activate pathways involved in cellular stress and inflammation. LDs are a pool of TAGs and their metabolism exerts regulatory effects on these pathways via releasing or removing signalling lipids [55]. As such, LDs are able to positively as well as negatively regulate lipid homeostasis. In this context, LDs can facilitate MUFA trafficking via PLIN5 and induce SIRT1 expression for exerting anti-inflammatory effects (for greater detail see Section 3.2.2) [56].

MUFAs can protect against saturated FAs induced apoptosis by binding to G-protein coupled receptors (GPCRs), especially GPR40, 41,43 and 120 as direct ligands [57]. MUFAs are also ligands of peroxisome proliferator-activated receptors (PPARs) and other nuclear receptors. They positively regulate the expression of genes responsible for mitochondrial and peroxisomal beta-oxidation. Furthermore, they inhibit pro-inflammatory nuclear factor (NF)-kB signalling, and thereby attenuate lipotoxicity [57,58].

Specific lipotoxic effects depend on the cell type and the molecular lipid species, its cellular localization and time of appearance. The wrong lipids at the wrong cellular location at the wrong time may cause detrimental cellular effects. FAs with less than 12 carbons can enter mitochondria without the carnitine shuttle, whereas long chain FAs depend on the system. Very long chain FAs (>22 carbons) undergo a preliminary beta-oxidation in peroxisomes, before the shortened FAs are further oxidized in mitochondria. Due to the close interaction of LDs with mitochondria and peroxisomes, LDs and the available FAs have a crucial role in the modulation of lipotoxicity [59].

Lipotoxicity can become self-escalating, as the metabolization of FAs at mitochondria and peroxisomes increases reactive oxygen species (ROS) formation. Elevated intracellular levels of ROS increase lipid peroxidation and ER stress and can induce apoptosis. Mainly the lysosomal pathway induces lipoapoptosis [51,60]. 

Lipotoxicity contributes to diseases including NAFLD, diabetes, neurodegenerative diseases as well as cancer. In hepatic steatosis (see Section 5.4), overabundant palmitate together with pro-apoptotic Bax can permeabilize the lysosomal membrane, followed by the release of the lysosomal cysteine protease cathepsin B into the cytosol. Then cathepsin B contributes to the promotion of mitochondrial dysfunction through the enhancement of t-Bid generation. Then, t-Bid induces the oligomerization of BAX and causes the permeabilization of the OMM. Here LDs can act in an anti-apoptotic fashion via their ability in storing overabundant palmitate as well as pro-apoptotic proteins like Bax [9,48]. 

The LD core acts as a storage depot for harmful lipid species including acylcarnitines (ACs), DAGs, and ceramides (CEs). ACs with medium- and long chain FAs are usually fueled into mitochondria as an energy source [61]. Overabundance of ACs impairs mitochondrial function, and thus increases levels of oxidative stress and apoptosis [62]. Consequently, elevated AC levels are associated with metabolic disorders like insulin resistance and cardiovascular diseases. Besides ACs, other mediators of lipotoxicity can be stored safely in LDs upon metabolization. 

The lipid composition of LDs including their buildup and breakdown products is a crucial step in the regulation of lipotoxicity [63]. Remodeling of LD proteins and lipids largely depends on COPI. COPI prevents full coverage of the LD surface by phospholipids [64]. Exposure of TAGs to the aqueous environment may prime LDs for organelle and protein interaction as well as binding events [65]. COPI also regulates the localization of key metabolic enzymes such as adipose triglyceride lipase (ATGL), glycerol-3-phosphate acyltransferase 4 (GPAT4) and DGAT2 to the surface of LDs [60].

Ectopic accumulation of specific lipids in liver and skeletal muscle cause metabolic derangements on several levels as exemplified for three molecular species:

(a) Free FAs (in particular saturated ones) can activate pro-inflammatory pathways by Toll-like receptor 4 (TLR4) mediated mechanisms of the innate immune system [66]. TLR4 activation strongly contributes to the development of NAFLD and Alzheimer‘s disease (AD) [67].
(b) DAGs and CEs are main molecular culprits involved in the development of insulin resistance [68]. DAGs activate protein kinase C (PKC) isoforms (among others PKCε), which phosphorylate the insulin receptor and inactivate its tyrosine kinase function.
(c) CEs activate other PKC isoforms (PKCζ) which interfere with AKT translocation and signaling downstream of the insulin receptor. Furthermore, CEs activate protein phosphatase 2A, which dephosphorylates and inactivates AKT signaling [69]. CEs negatively affect the permeability of the OMM and form channels for the release of pro-apoptotic proteins in the mitochondrial intermembrane space. The pro-apoptotic protein Bax stabilizes the channel [70]. Different Cer species within a cell have specific and diverse effects on the translocation of Bax to the OMM. There is evidence that excess CEs need to be stored in LDs. The conversion of CEs to acylceramides is catalyzed by DGAT2 [71], and thus storage in LDs may have a protective function (see Figure 1). Failure in the production of omega-O-acylceramides occurs in neutral lipid storage diseases (NLSD).

Besides CEs, PUFAs such as intracellular arachidonic acid (AA) can induce apoptosis via activation of caspase 3. To avoid cellular toxicity of non-esterified AA, cells can catabolize it by cyclooxygenase-2 (COX-2), LOX and FA-CoA ligase 4 (FACL4). The upregulation of pathways that activate these enzymes blocks apoptosis. Importantly, AA can be stored in LDs during inflammation with a concomitant reduction of apoptotic rates [55]. In addition, important mediators can be produced non-enzymatically by the interaction of lipids with ROS [72,73]. Bioactive lipids serve as precursors of specialized oxidative products during cell or tissue oxidative stress. These highly bioactive mediators specifically include prostaglandin-derived isoprostanes and arachidonic acid-derived isoketals. Rising evidence suggest that ROS-generated isoprostanes act as pro- or anti-inflammatory mediators in many diseases [72,74]. In detail isoprostanes extensively contribute to inflammatory processes in atherosclerotic cardiovascular diseases [75,76], neurodegenerative diseases [77,78] and NAFLD [79]. For further details on the role of LDs in inflammation see Section 5.5.

Lipid peroxidation is closely linked to apoptosis and lipotoxicity and PUFAs are highly susceptible for the process. Finally, hydroxyl radicals arise, which cause lipid peroxidation of FAs in lipoproteins and membranes. LDs can prevent lipid peroxidation at an early stage and store lipid peroxides to prevent further damage [80]. Lipid peroxidation contributes to the development of many diseases including NASH. Recent findings in yeast additionally underline the importance of LDs for detoxification of damaged as well as pro-apoptotic acting lipids [81]. Not only proteins and lipids but also hydrophobic xenobiotics can be safely stored in LDs [65,82].

### 3.2. Protein Homeostasis

#### 3.2.1. The Fate of Misfolded Proteins

Protein homeostasis is of utmost importance for cellular integrity. During the folding process, proteins acquire a structure that is associated with minimal free energy and is thermodynamically stable. Sometimes proteins fail to fold correctly and gain a false conformation [83,84], and mutations as well as stressful situations increase misfolding. ROS originate from cellular metabolism or extracellular sources and can react with amino acids including histidine, leucine, methionine, and cysteine. These oxidative modifications either alter enzyme activity or result in protein unfolding. As another consequence, the formation of stable carbonyls can induce conformational changes in the polypeptide chain (for a detailed review see [85]. Cells use highly effective mechanisms to remove potentially dangerous proteins. An active and cytoskeleton dependent process translocates such proteins to distinct cellular aggregates. These aggregates are best described in yeast cells and include the JUxtaNuclear Quality Control Compartment (JUNQ; nuclear localization), the IntraNuclear Quality Control Compartment (INQ; nuclear localization), the Insoluble PrOtein Deposit (IPOD; phagophore assembly site localization close to the vacuole), as well as cytosolic Q-bodies (CytoQ) [86,87,88,89]. The fate of misfolded and damaged proteins at the aggregation sites differs. The presence of the molecular chaperone HSP104 at protein aggregates promotes refolding attempts [90,91]. The IPOD is mainly composed of misfolded proteins with low solubility and appears in stressed as well as unstressed cells. Its close proximity to the vacuole indicates the removal via autophagy [92]. JUNQ, INQ and CytoQ aggregates indicate cellular stress and harbour misfolded and aggregated proteins with some higher solubility [89]. The sorting signal for JUNQ is ubiquitination [87], and 26S proteasomes degrade terminally misfolded proteins [86,91]. A wide variety of diseases evolve due to the presence of such aggregates (e.g., amyloidosis and prion diseases). Under conditions of severe proteotoxic stress, LDs and mitochondria assist in removing of such aggregates as we will show in the following section (see Figure 1). Growing evidence also suggests a pivotal role of aggregates and LDs in neurodegenerative disorders [77]. Further details will be presented in Section 5.1.

#### 3.2.2. LDs As A Protein Docking Site

When the proteasomal system is overwhelmed under severe stress conditions LDs have a supportive role. LDs and protein aggregates come into close proximity upon heat stress. Cells devoid of LDs and defective in sterol biosynthesis cannot effectively remove protein aggregates. LDs may supply the aggregates with sterol derivatives, which act as solvent and are crucial for the clearance of misfolded proteins [93].

Mitochondria also contribute to the removal of misfolded proteins. Deletion of the cytosolic chaperone Hsp70 results in the relocalization of misfolded proteins from the cytosol to the matrix of mitochondria. Degradation of misfolded proteins ensues at the matrix resident LON protease instead of the proteasome. This degradative process is known as MAGIC (mitochondria as guardian in cytosol) and may be supported by LDs. Mmi1 is a marker protein of MAGIC degradation and shuttles from mitochondria to LDs upon stress induction. In addition, certain pro-apoptotic proteins may shuttle from the cytosol to mitochondria. They have the capacity to permeabilize the OMM and can be removed by LDs after stress. Subsequently, these protein containing LDs reach the vacuole for degradation by lipophagy [9].

LDs can also remove misfolded proteins from the ER. Misfolded proteins in the lumen of the ER are ubiquitinated by ER resident enzymes, translocated back to the cytosol and are then degraded by the 26S proteasome. This pathway is known as ERAD (ER-associated (protein) degradation) [94]. The antibiotic tunicamycin is an agent that blocks the first step in the glycosylation of proteins and leads to unfolded proteins in the ER lumen [95]. ER stress initiation by tunicamycin also dramatically stimulates the number and size of LDs in the cell [96]. LDs specifically form at sites of stress induced ER aggregates that are marked by the chaperone Hsp104, and these LDs harbour polyubiquitinated proteins [97]. LDs may therefore assist the 26S proteasome during stress in the removal of damaged, unfolded and potentially dangerous proteins. Accordingly, a specific blockage of the proteasome by the inhibitor MG132 increased intracellular LD numbers [98]. Accumulating LDs are subsequently removed by the cell through microlipophagy [97]. The removal of unfolded proteins from the ER by LDs closely resembles the cytosolic and mitochondrial pathways. It is of interest that several components of ERAD, such as UBXD2, VCP, AUP1 and SELENOK are present in the human LD proteome, indicating a general mechanism of protein homeostasis [99]. More than 60 diseases are based upon defects in ERAD [100] and a considerable number of them show either an accumulation of LDs, LD malformations or altered LD distribution (e.g., [101,102,103,104].

Besides unfolded proteins, viral proteins occur at LDs and viruses can hijack LDs for their assembly. In addition, certain cellular proteins with regular conformation use LDs as a platform for storage and redistribution. Histones H2A, H2B and H2Av connect to LDs of oocytes in *Drosophila melanogaster*. This is of special importance for the growing embryo, as rapidly synthesized DNA needs to be packaged by histones. In this special scenario, LDs close at the nuclei act as a deposit for histones [105]. LDs can also influence the expression of certain genes. The transcription factor NFAT5 (Nuclear factor of activated T-cells 5) resides in the cytosol and translocates to the nucleus upon application of hyperosmotic stress. Upon binding to response elements on the DNA, an adaptive cellular response, which includes the production of osmolytes and expression of Hsp70 is initiated [106]. In adipocytes, LDs can attach to the cell death inducing DFFA like effector c (CIDEC) that among other functions promotes the growth of LDs. Yeast-2-hybrid analysis revealed a physical interaction between CIDEC and NFAT5. In fact, overexpression of CIDEC sequesters NFAT5 to LDs and thus prevents a stress induced translocation of NFAT5 to the nucleus and a proper stress response [107].

Expression levels of PLIN5 are high in tissues with augmented oxidative ATP production in mitochondria such as skeletal muscle, heart and liver. PLIN5 rich LDs physically interact with mitochondria [108], but PLIN5 was also found in the cytosol, at the ER and the nucleus. Starvation or catecholamine treatment induces nuclear localization and phosphorylation of PLIN5. In the nucleus, PLIN5 interacts with the transcriptional coactivator PGC-1α (peroxisome proliferator-activated receptor gamma coactivator 1-α) and the NAD^+^-dependent deacetylase SIRT1 (sirtuin 1). Stimulated activity of SIRT1 induces the deacetylation and activation of PGC-1α [109]. PCG1-1 is deeply involved in the biogenesis of mitochondria and respiration [110]. Several tissue specific isoforms of PGC1alpha may increase the complexity of the interaction, and some brain isoforms are associated with neurodegenerative diseases [111,112,113]. Therefore, PLIN5 can influence mitochondrial activity in several ways, either by mediating an interaction with LDs or by altering gene expression. Furthermore, LDs indirectly modulate the general stress response as well as inflammation via the PLIN5-SIRT1 axis [114].

#### 3.2.3. Protein Motifs for LD Localization

**Figure 2:**
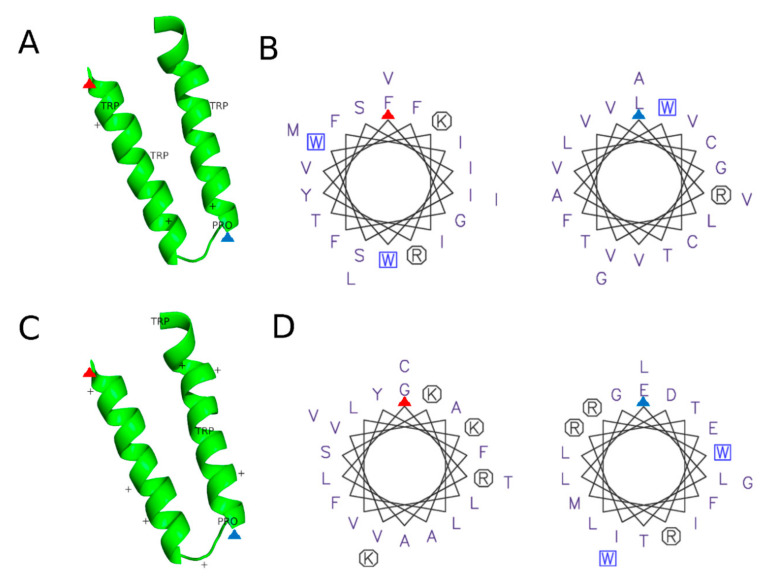
(**A**) Structure prediction of LiveDrop is based on the residues 163–206 of Drosophila GPAT4 (isoform B) [64] mounted on the v-shaped hairpin motif of the BAX protein [9] so that proline residues are aligned (residues 108–151; coordinates from PDB 1f16, chain A). Labels show proline, positively charged residues (H, K, R) and tryptophans. In (**B**) Helical wheel plots for the N-terminal helix (residues 163–184) and C-terminal helix (residues 186–206) of LiveDrop are presented. Positively charged residues are shown in octagons, tryptophans in squares (EMBOSS pepwheel). The first amino acid in helix one is marked by a red triangle, the first amino acid in helix 2 is marked by a blue triangle. Amino acids 1–18 are located in the inner circle, amino acid 19 and the following in the outer circle. (**C**) shows a structure of the v-shaped hairpin motif of the BAX protein. Labels as in (**A**). In (**D**) Helical wheel plots for the N-terminal helix (residues 108-129) and C-terminal helix (residues 131–151) of the BAX hairpin are displayed. Symbols as in (**B**).

Some proteins cannot reach LDs, as certain requirements are prerequisite for LD localization. [9]. LD proteins can enter either from the ER or from the cytosol. Accordingly, two distinct classes of LD resident proteins, namely Class I and II proteins can be distinguished [115]. Class I proteins are synthesized and inserted into the membrane at the ER. These proteins transfer via the lipid bridge that forms between the ER and the LD [64], and typical examples are GPAT4 and DGAT2. No classical consensus sequence or motif for LD localization has been reported so far, but some features for membrane insertion and shuttling start to emerge (Figure 2). Most of these proteins contain a hydrophobic, v-shaped hairpin sequence (two α-helical domains with a central proline, or “proline knot”). In many cases, positively charged amino acids with (a) hydrophobic tryptophan(s) in the middle flank the helices [116,117,118]. Most cytosolic class II proteins, such as PLINs target LDs with amphipathic helices or short hydrophobic sequences [115]. Due to their monolayer membrane, the surface of LDs is prone to phospholipid packaging defects. Proteins with amphipathic helices, such as CTP: phosphocholine cytidylyltransferase [118] are attracted by these imperfections and specifically attach to the surface of LDs [119]. All PLINs (PLIN1-PLIN5) contain several 11-mer repeats, which form amphipathic helices that have the capacity to bind LDs. Point mutations in these regions inhibit the localization to LDs [120,121].

Potentially harmful proteins, such as the mitochondrial anti-apoptotic TCTP, as well as pro-apoptotic BAX can relocalize to LDs A v-shaped hairpin structure in the BAX protein is required for the transfer process (Figure 2C). A GFP tagged version of the protein domain is sufficient to induce both, mitochondrial and LD localizationThe v-shaped hairpin in BAX closely resembles a 56 amino acid long hairpin domain in GPAT4, termed LiveDrop, which is required for LD localization (Figure 2C). The LiveDrop domain contains characteristic hydrophobic helices (Figure 2D) that contain a central tryptophan and positively charged amino acids at the hinges (Figure 2C). In the loop region, a proline residue causes a kink. Most elements are present in the BAX hairpin structure (Figure 2A,B). Due to its capacity to modulate apoptosis by redirecting proteins from the OMM, LDs have the capability to modulate diseases associated with alterations in apoptosis (e.g., cancer; several neurological disorders; several cardiovascular and autoimmune diseases) [122].

## 4. The Role of LDs in Cellular Stress and Aging

### 4.1. The Function of LDs in Modulating Stress

Different organelles have overlapping and diverse responses to cellular stressors. Stress response of mitochondria includes a widespread reconstruction of the mitochondrial network. In the first step, mitochondria execute hyperfusion to supply the cell with increased amounts of ATP for energy consuming stress response. Upon prolonged stress, the tubular network completely fragments. The fission process is a prerequisite for either OMM perturbation or the removal of damaged mitochondria via autophagy [123,124].

LDs stress responses differ to those of mitochondria and depend on LD size. In general two major subtypes of LDs can be distinguished as ER-associated LDs and cytosolic-LDs [125]. Large, ER-associated LDs are in constant contact with the ER and receive persistent lipid supply via the lipidic bridge. After detachment from the ER and disruption of the lipidic bridge, LDs decrease in size and transform into cytosolic LDs [126]. Furthermore, LDs do not equally distribute in healthy cells, but occur in clusters that are in close proximity to the nucleus [127]. It is possible that such LDs are ER-associated LDs that constantly bud off and are fed by the ER. Starvational stress induces LD redistribution and increased motion of the organelles. LDs move from the center of the cell to the periphery, where they get in close contact with mitochondria. The process is downstream of AMPK (AMP-activated protein kinase) activation that promotes LD dispersion on detyrosinated microtubules to increase mitochondrial FA oxidation [127]. Besides nutritional stimuli, physical exercise, stress and mitochondrial fragmentation induce the interaction between mitochondria and LDs [9,108,128]. The inter-organelle communication is preserved from yeast cells to mammals [37]. Some cell types (e.g., adipocytes) show profound interaction of mitochondria and LDs, and therefore these mitochondria are called “peridroplet mitochondria” [108]. A hallmark of stress is an increase in LD formation, as observed in a broad variety of cell types [9,80,96,129]. Several mechanisms cause the increase in LD numbers and inter organelle communication:

(a) The high energy demand during stress response causes fueling of FAs from LD to mitochondria for β-oxidation [130]. Reverse transfer of lipids occurs, and lipid relocation from mitochondria to LDs may protect from lipotoxic settings [131] and aberrant lipid signaling (see Section 3.1).
(b) LDs are involved in protein homeostasis and assist in the removal of damaged and misfolded proteins from mitochondria (see Section 3.2)
(c) LDs modulate the apoptotic program by redirecting acylcarnitines from mitochondria (see Section 3.1). Similarly, mitochondrial proteins involved in the apoptotic program translocate [9,81]. In several studies, the pro-apoptotic protein BAX was found as a *bona fide* member of the LD proteome [9,99,132].
(d) LDs fulfill antioxidative functions, reduce ROS levels and prevent the peroxidation of PUFAs. The mechanism involves the removal of these lipids from membranes to the core LDs, where they are less accessible to ROS [129]. Consequently, a stimulated increase in LD number can augment cell survival upon the onset of stressors [9].

Finally, cellular stress induces a changed lipid as well as proteome composition in LDs. In general, levels of proteins and lipids at LDs increase, indicative for their important role as a lipid as well as protein buffer [81]. Stress response induced alterations of LDs resembles changes that occur during cellular aging [81]. A prominent aging theory is the “mitochondrial free theory of aging” (MFRTA) proposed by Denham Harman in 1972 [133]. The electron transport chain (ETC) at the inner mitochondrial membrane is a prerequisite for the formation of the proton motive force and ATP production. The premature leakage of electrons at complex I and III causes the formation of superoxide [134]. MFRTA claims that ROS levels dramatically increase with aging and react with organic components of the cell, including DNA, proteins and lipids. In line with this, stress as well as aging induces the accumulation of LDs in yeast and mammalian cells [135,136,137]. There is growing evidence that LDs play a role in the regulation of longevity [138]. Results from the yeast *S. cerevisiae* strongly indicate the involvement of LDs in cellular aging. In yeast cells, two forms of aging processes, namely chronological and mother-cell specific (replicative) aging occurs [139,140]. Chronological aging that resembles aging in human differentiated cells reduces the viability in stationary phase when all nutrients are exhausted. Replicative aging that mimics the Hayflick limit in stem cells describes the mechanisms of asymmetric budding (the mother cells age and the daughter cells rejuvenate themselves). The deletion of either the diacylglycerol acyltransferase *DGA1* or the Acyl-CoA:sterol acyltransferase *ARE1* and *ARE2* shortens the chronological lifespan. Both interventions reduce TAG as well as sterol ester levels and the number of LDs. Interestingly, the deletion of the TAG lipases TGL1-5 prolongs the chronological lifespan [141].

### 4.2. Emergence and Role of LDs During Aging

Since 1935 caloric restriction is a known and effective intervention to extend lifespan in rodents [142]. This finding holds true for many model organisms [143,144,145]. Caloric restriction initiates a metabolic change towards fatty-acid synthesis. Furthermore, caloric restriction enlarges LDs as well as influences the expression of genes involved in lipid metabolism, which is crucial for the life prolonging effect [146,147].

LDs may also affect longevity in mammals, as they can activate the sirtuin SIRT1. In many studies and in many model organisms, activation of this enzyme leads to a prolonged life, improved organ functions, a higher fitness and increased stress and disease resistance [135].

Several studies report a positive effect of olive oil on health and lifespan. This could partly explain the “French paradox”, the paradoxical epidemiological finding of a low incidence of coronary heart disease despite a diet rich in saturated FAs [148]. The most prevalent FA in olive oil is the MUFA 18:1 oleate (up to approximately 80%). Feeding of rats with olive oil increases their lifespan to 157% [149], and feeding mice with olive oil decreases body weight as well as fat mass [56]. Oleate generally stimulates LD formation [150], but hepatic LDs adapt their size from large to small, but PLIN5-rich LDs [56]. PLIN5 translocates into the nucleus upon binding of MUFAs, interacts with SIRT1 and PGC-1α, and induces the expression of genes such as UCP1, CPT1α, and complex I-IV of the respiratory chain. Consequently, oxygen consumption as well as heat production is increased [56]. Growing evidence suggests that altered lipid signaling could promote replicative senescence in yeast. It was shown that the amount of 19 polyunsaturated TAGs significantly changes during the aging process [151,152]. On the other hand, increased LD number is not always beneficial as some diseases and pathologies are associated with higher LD abundance, such as NLSD and atherosclerosis (for a detailed review see [153]). Therefore, balanced LD turnover is of utmost importance and strictly depends on the organism, tissue, cell types and growth phase of the cell. In the following, we will summarize selected diseases and pathologies that originate from imbalances in LD metabolism.

## 5. The Role of LDs in Disease

### 5.1. LDs and Neurodegenerative Diseases

Neurodegenerative diseases are associated with toxic protein accumulation, mitochondrial defects and elevated ROS levels that result in the decay of neurons (see Figure 1). Interestingly, these stressors also cause the accumulation of LD in glial cells [154]. One prevalent neurodegenerative disease with a close link to LDs is the neurodegenerative movement disorder Parkinson disease (PD). Defects in the proteins that connect the OMM with LDs, such as mitochondrial MFN2 and the LD resident PLIN1 have a role in the development of PD [33].

PD is characterized by the loss of dopaminergic neurons in certain areas of the substantia nigra. In PD, dysregulated accumulation of α-synuclein (aSyn) in the cytoplasm of distinct neurons occurs [155]. The accumulation is a multistep process and after the oligomerization of aSyn into small protofibrils, insoluble large aSyn fibrils are formed [156]. The N-terminal amphipathic region of aSyn enables binding to lipid membranes. This domain contains multiple lysine residues that contribute to electrostatic interactions with negatively charged membrane surfaces [157]. Strikingly, the LD monolayer attracts aSyn, where it preferentially accumulates [158]. PD patients additionally upregulate key enzymes of phospholipid metabolism in the substantia nigra, including phospholipase A2, phosphocholine cytidyltransferase and phosphoethanolamine cytidyltransferase [159]. Similarly, imbalances in aSyn homeostasis have severe effects on lipid metabolism. Toxic aSyn overexpression increases levels of DAGs, TAGs as well as oleate [160]. LD upregulation in PD may provide a “sponge/sink” for toxic aSyn proteins. Concomitant with that the role of LDs is ambiguous, as increased levels of LDs and Plin4 cause reduced mitophagy in mouse neurons and promote PD progression [161]. Reduction of LD formation is crucial for the toxicity of aSyn in yeast and induces apoptosis [160].

LDs are also associated with the development of additional neurological disorders such as Huntington’s disease (HD) and Alzheimer disease (AD). LDs are detectable in brain sections of HD models [162]. Changes in brain cholesterol homeostasis in HD increase the accumulation of cholesterol in striatal neurons [163], resulting in neuronal dysfunction [164]. In a HD model in Drosophila melanogaster, changed cellular lipid metabolism associates with altered LD size [165]. HD development largely depends on a CAG repeat expansion in the exon 1 of huntingtin gene, encoding polyglutamine repeats in the protein. The mutated huntingtin protein was associated with increased LD numbers in mouse models as well as cells derived from HD patients. Human striatal tissue of HD patients also shows increased Oil Red O staining intensities [162].

AD is the most common neurodegenerative disease and associated with microglia mediated inflammation and alterations of lipid metabolism. Already Alois Alzheimer described “adipose saccules” in many glial cells [166], but the discovery was ignored for almost a century. LD formation increases in murine and human AD samples and correlates with dysfunctional neurogenesis [167]. The connection between LDs and AD became also evident by findings on protein-O-linked hexosaminidase C (also known as N-Acetyl-β-D-glucosaminidase). Hexosaminidase C accumulates on the surface of nascent, PLIN2 containing LDs. The enzyme acts as a regulator of LD formation and pharmacological inhibition of hexosaminidase C slows neurodegeneration in various AD mouse models [168]. Imbalances in lipid metabolism and LD function play a major role in the development of AD [169]. Some data strongly suggest that LD functions may have an important role in development of AD, although the precise roles are yet unclear.

The accumulation of cholesterol and CEs in neurons is associated with neurodegeneration [170]. Furthermore, altered sphingolipid metabolism and their accumulation in the brain links to AD development [171,172]. In addition to neurons, glial cells and alterations in their lipid metabolic functions contribute to the development of neurodegeneration. The APOE E4 allele is a strong genetic risk factor for late onset of AD [173], while the APOE E2 allele lowers risk [174] in comparison to the common APOE E3 allele. APOE is a component of low-density lipoprotein (LDL) particles and astrocytes with E4 expression have small LDs compared to E3 expressing cells [77]. Astrocytes expressing E4 have increased PLIN2 levels and less efficient uptake and oxidation of FAs. Other glial cells contribute to the development of AD, and the activation of microglia and associated inflammatory processes are commonly present. Microglia also are becoming gradually activated and apparently dysfunctional during the ageing process. The activation of microglia includes the accumulation of LDs, in a process similar to the formation of foam cells in atherosclerosis. We recently reported the build-up of LDs in microglia in aging murine and human brains and denominated theses sells as LDAM (LD accumulating microglia) [175]. Mouse hippocampus microglial LDs contained PLIN3, whereas PLIN2 was present in microglia in human hippocampal brain sections and more abundant in aged individuals. The accumulation of LDs causes less efficient phagocytosis, higher levels of ROS and increased secretion of pro-inflammatory cytokines. LDAM have a unique transcriptional profile that supports inflammation [175].

Another disease featuring neurological decline is chorea acanthocytosis. This hereditary disease is exceptionally rare and caused by a mutation in the ER-LD connective protein VPS13A gene (see Section 2.2). Chorea acanthocytosis is an autosomal recessive disease, histologically characterized by acanthocytes, a pathological form of erythrocytes and shares similarities with HD [176].

Mutations in ER-LD tether protein SNX14 are associated with autosomal recessive Spinocerebellar Ataxia 20. The neurodevelopmental disorder belongs to a large heterogeneous group of spinocerebellar ataxias. Characterized by an early onset during infancy, the disease causes cerebellar atrophy and severely impaired psychomotor development [31].

Failure in the transfer of FAs from LDs to peroxisomes for beta-oxidation causes neuronal diseases such as spastic paraplegia and adrenoleukodystrophy. Mutations in the SPAST gene cause spastic paraplegia 4, an autosomal dominant and most prominent form of hereditary spastic paraplegias. The resulting protein product encodes an AAA ATPase involved in the severing of microtubules. Hereditary spastic paraplegias are generally defined by progressive spasticity and weakness in lower extremities [177]. Mutations in ABCD1, a member of the ABC transporter family cause the accumulation of very long chain FAs in the cell in adrenoleukodystrophy [178]. ABCD1 resides in the peroxisomal membrane and upon dimerization facilitates trafficking of very long chain FAs into peroxisomes. Failure of this transport severely damages the myelin sheath in neurons and may ultimately cause seizures, persistent vegetative state and death [31]. These genetic data strongly suggest that LD functions may have a causative role in development of neurodegenerative diseases.

### 5.2. Neutral Lipid Storage Disease (NLSD) and Rare Lipid Storage Diseases

NLSDs are rare autosomal recessive genetic disorders without specific therapies, and are associated with the accumulation of LDs in different cell types. Mutations occur either in adipose ATGL (encoded by PNPLA2) [179], or in α,β-hydrolase domain 5 (ABHD5, also known as CGI-58) [180], two genes with key functions in LD metabolism. NSLD phenotypes are characterized by the manifestation of either myopathy or ichtyosis [181]. NLSDs with myopathy develop progressive myopathy, cardiomyopathy, hepatomegaly, chronic pancreatitis and diabetes [182,183,184]. Large genetic heterogeneity in ATGL mutations causes wide variations in clinical phenotypes. The mutations affect either the binding to LDs or the catalytic activity of ATGL. Mutations in ABHD5 cause NLSDs with ichthyosis and additionally affect other organs, including liver, skeletal muscle, CNS and eyes [185]. ATGL activation is promoted by ABHD5, although ATGL mediated hydrolysis of TAG occurs in the absence of ABHD5 at low efficiency [186]. Lipolysis is a highly regulated process and ATGL catalyzes the rate-limiting step, followed by hormone sensitive lipase and monoacylglycerol lipase. FAs can be a substrate for mitochondrial beta-oxidation, synthesis of lipid mediators such as leukotrienes or membrane lipid synthesis. ATGL interacts with various cell-type enriched inhibitors or activators, frequently phosphorylated upon stimuli. In the context of NLSDs, adipocyte-enriched PLIN1 sequesters ABHD5 on the LD surface and inhibits lipolysis. Protein kinase A mediated phosphorylation of PLIN1 causes the release of ABHD5 and subsequent interaction with and activation of ATGL. In skeletal and cardiac muscle, the phosphorylation of PLIN5 similarly promotes lipolysis [1]. In NLSDs, hampered TAG degradation reduces the availability of FAs and causes the cytoplasmic accumulation of TAGs in many tissues. Consequently, the staining of neutral lipids in peripheral blood granulocytes confirms the clinical diagnosis [187,188]. In NLSDs with skeletal muscle myopathy, LD number increases in mainly oxidative muscle fibers between myofibrils and probably cause lipotoxic effects [189,190]. The accumulation of LDs in the heart mainly occurs in cardiomyocytes, but also affects endothelial cells, smooth muscle cells and intima media foam cells [191,192].

In NLSD forms with ichthyosis, the liver is frequently affected and manifestations include hepatomegaly and steatosis in more than 80% of cases. Liver biopsies reveal the accumulation of large LDs mainly in hepatocytes [193,194]. A common finding in NLSD forms with ichthyosis is also the accumulation of LDs in skin fibroblasts and keratinocytes [195]. ABHD5 also activates PNPLA1, which catalyzes the final step in omega-O-acylceramide biosynthesis [196]. Usually, Omega-*O*-acylceramides are essential for skin permeability and its barrier function [197]. Mutations in ABHD5 may therefore specifically cause the ichthyosis by the reduced availability of these lipids [198].

In contrast to NLSD, lipodystrophy is characterized by the inability to store TAGs in adipose tissue. Several genes participating in LD biology influence lipodystrophy. Some regulate *de novo* TAG synthesis like 1-acylglycerol-3-phosphate *O*-acyltransferase 2 (AGPAT2) and lipin1 (LPIN1). Others include the seipin BSCL2, which is a mayor-contributing factor in LD biogenesis [135,199].

Another disease manifestation associated with the loss of adipose tissue and therefore LDs is cachexia. Cachexia is a common comorbidity of cancer patients and manifests as an imbalance between catabolic and anabolic processes. Particularly changes in lipid metabolism cause rapid loss of adipose tissue and skeletal muscle [135,200]. The exact molecular connections between LDs and cachexia are yet unknown. *Atgl* knockout protects mice from skeletal muscle loss, and therefore increased lipolysis seems important for the pathophysiology of cachexia [201].

### 5.3. Atherosclerosis

Atherosclerosis is characterized by an accumulation of lipids and fibrotic material in the wall of large arteries. The accumulation of macrophages and their transition into LD-rich “foam cells” is a central component in development of atherosclerotic plaques. In addition to macrophages, smooth muscle cells can also convert into foam cells with large LDs [202,203]. Recent evidence suggests that up to 70% of foam cells in mouse atherosclerotic lesions originate from smooth muscle cells [204]. Such smooth muscle cell derived foam cells also significantly contribute to and are present in human lesions [205]. The formation of plaques in arterial walls occurs during all stages of atherosclerotic development and causes major cardiovascular diseases and stroke. Cytosolic LDs accumulate in foam cells and are rich in CEs, which originate from modified LDL particles. In addition, human atherosclerotic plaques also contain oxidized sterols and non-sterol lipid species [206]. Oxidized LDL stimulated a strong induction of isoprostane generation in human macrophages and LDs in foam cells may provide a storage site to prevent toxic consequences [207]. Macrophages take up oxidized LDL via scavenger receptor mediated endocytosis and other mechanisms [208]. Lysosomal acid lipase (LAL) hydrolytically cleaves LDL derived CEs into free cholesterol and FFAs. Free cholesterol may efflux from cells via ATP binding cassette (ABC) transporters or be re-esterified to FAs in the ER prior to its storage in LDs. Neutral cholesterol ester hydrolases can associate with LDs and mobilize stored CEs (for further details on CEs see Section 3.1). Hydrolysis of these CEs probably depends on several hydrolases, including LD-associated hydrolase that is highly abundant in human macrophages and atherosclerotic lesions [209]. Alternatively, LDs degradation follows autophagy and fusion with lysosomes prior to CE hydrolysis [210]. Free cholesterol efflux in macrophages occurs via the ABC transporters ABCA1, ABCG1 and ABCG4 [211], and transfer to high density lipoprotein (HDL) is an integral part of reverse cholesterol transport (RCT). RCT seems atheroprotective, and therefore increased hydrolysis of cholesterol-esters in LDs may be a therapeutic target. Foam cells derived from smooth muscle cells decrease their ABCA1 expression in comparison to macrophage derived foam cells, which probably contributes to less efficient cholesterol efflux [205]. Lipid acceptors, such as lipid poor apoA-I cannot only accept cholesterol, but also accept other lipid species from LDs of foam cell [212]. The expression of PLIN1 in macrophages remains controversial [213], but PLIN2 and PLIN3 are abundant in foam cells and atherosclerotic plaques [214,215]. PLIN2 is highly expressed in macrophages, and its expression increases upon lipid loading [216]. Mice with deleted PLIN2 exhibit decreased lesion formation in the ApoE knockout background [215]. PLIN2 deficiency does not increase cholesterol-associated toxicities in macrophages [217]. Furthermore, PLIN4 and PLIN5 expression is upregulated in THP-1 macrophages upon treatment with oxidatively damaged LDL [216]. The same study identifies LD proteins CIDEA, CIDEB and CIDEC (cell death activator proteins) in macrophages, although their contribution to atherosclerosis remains unclear. Foam cells also generate pro-inflammatory signals within the arterial wall. PLIN2 overexpression increases the secretion of chemokines and cytokines such as IL-6, TNF-alpha and MCP-1 [218]. The role of PLIN1 in the development of atherosclerosis remains unclear with conflicting evidence [213,219]. Overall, LDs have a causative role in the development of atherosclerosis.

### 5.4. Obesity and Non-Alcoholic Fatty Liver Disease

Obesity often associates with non-alcoholic fatty liver disease (NAFLD), and the chronic liver condition affects approximately 30% of the population in Western countries [220]. NAFLD comprises the accumulation of LDs in liver tissue and covers a spectrum from non-alcoholic fatty liver (NAFL) and non-alcoholic steatohepatitis (NASH) to fibrosis, and finally cirrhosis. Cirrhosis is an important risk factor for hepatocellular carcinoma (HCC) [221], one of the most frequent malignancies on a global scale. HCC has a poor prognosis and is the third most frequent cause of cancer deaths worldwide [222]. In NASH, significant inflammation and some scarring complements NAFL. Obesity and associated metabolic diseases frequently share insulin resistance as a common basis. NAFLD is thus widely considered as the hepatic manifestation of the metabolic syndrome and insulin resistance [223]. Excessive fat storage in LDs of hepatocytes and non-parenchymal liver cells promotes lipotoxicity and cellular damage. Attracted and activated Kupffer cells release ROS and other signals, which stimulate additional inflammatory cells. During NASH progression, lipid peroxidation and ROS augment liver steatosis by enhancing LD formation [224]. Kupffer cells activate stellate cells (also known as Ito cells) from a quiescent, vitamin A storing phenotype [225] into proliferating, fibrogenic and contractile myofibroblasts [226]. Strong evidence suggests that the accumulation of LDs and their metabolism contribute to NAFLD. Interestingly, LDs of stellate cells [227], Kupffer cells and hepatocytes contribute to NAFLD pathophysiology and sequelae [228]. A hallmark of NAFLD is the development of particularly large LDs in hepatocytes. The formation of giant-sized LDs arises from either coalescence or from facilitated diffusion of lipids from one LD to another [229]. Distinct proteins accumulate during NAFLD on the surface of LDs. One example is PNPLA3 (also known as adiponutrin), which encodes a TAG lipase homologous to adipose triglyceride lipase. The physiological roles of PNPLA3 are unclear, but a gene polymorphism is the predominant genetic risk factor for NAFL and its progression [230]. Additionally 17β-hydroxysteroid dehydrogenase 13 contributes to sex steroid synthesis, binds to LDs and promotes the development of NAFLD [231,232]. Other LD-specific proteins, such as CIDE proteins [233,234] and several PLINs also accumulate during NAFLD [228,235]. Members of the CIDE protein family promote larger LDs [236,237]. PLIN1 is present on LDs during the development of NAFL [238,239]. PLIN2 is strongly upregulated in NAFLD [239] and inhibits FA oxidation [240]. PLIN3 is upregulated in mice on high fat diet and reduction of PLIN3 levels improves NAFLD [241]. In addition, PLIN5 expression strongly increases in mouse NAFL [242]. High levels of PLIN5 can specifically recruit and attach LDs to mitochondria [108], as already discussed above. Emerging evidence suggests that mitochondria-LD interactions play critical roles in NAFLD pathogenesis and progression [243]. Mitochondria bound to LDs have distinct fusion-fission properties, enhanced bioenergetics capacities and support the expansion of LDs [244]. The direct interaction of mitochondria with LDs provides lipids for energy production via mitochondrial β-oxidation. The contact sites depend on PLIN2 and PLIN5, which additionally modify the efficiency of FA oxidation [240,245]. With the onset of hepatic steatosis, increases in mitochondrial FA oxidation, tricarboxylic acid cycle activity and oxidative phosphorylation ensue [246]. Increased mitochondrial activity initially seems to protect hepatocytes from lipotoxic effects of FFAs [246]. On the other hand, higher metabolic fluxes in mitochondria also increase ROS generation. ROS mediated damages intensify hepatic lipid accumulation, trigger inflammation and fibrosis, and cause cell death. LDs and their direct interaction with mitochondria also regulate apoptosis and cell death in NAFL. Hepatocellular death is a hallmark of NASH and reported for in vitro models and patients [247]. Higher apoptosis rates at earlier stages of NAFLD ensue prior to a reduced onset of apoptosis in HCC. Additional types of programmed cell death, such as pyroptosis partially overlap with classical apoptosis in NAFLD [248]. Increased levels of pro-apoptotic Bcl2 proteins, such as BAX and BAK appear in NASH [249]. On the other hand, expression levels of anti-apoptotic proteins such as Bcl-XL are reduced [247]. With the progression to HCC, rates of apoptosis strongly decrease and carcinoma cells become insensitive to the induction of apoptosis. The over-expression of anti-apoptotic Bcl-XL, MCL-1, XIAP and survivin seems to block the induction of apoptosis [250]. Specific proteins transfer from the ER to LDs through membrane bridges [64]. As such, LDs can specifically remove oxidatively damaged and aggregated proteins from the ER. Misfolded proteins also accumulate in inclusion bodies and LDs are essential for their efficient clearance [93]. In a recent work, we described a mechanism, by which damaged and apoptosis-related proteins relocate from the OMM to LDs in yeast and human hepatoma cells. The process probably promotes mitochondrial rejuvenation [9]. Others confirm the finding that BAX can also localize to LDs [99,132]. Additionally, mitochondria can transfer lipids to LDs to avoid lipotoxicity and aberrant lipid signaling [131]. An altered LD lipidome may also support the insertion of specific proteins. The size and curvature of LDs seems important for interactions with other organelles, as giant sized LDs typically develop in NASH [229], but LD size decreases during cirrhosis and progression to HCC [251]. Small LDs preferentially contain PLIN5, which facilitates the interaction with mitochondria [120]. Toxic lipids and proteins then shift via LDs to lysosomes, where degradation via lipophagy occurs. LDs in various hepatic cell types are crucial components in NAFLD pathophysiology.

### 5.5. LDs and Inflammation

LDs regulate the availability of FAs and derivatives thereof, which are essential for physiological signaling pathways. LD turnover increases during nutritional, metabolic and oxidative stress [80,81,252]. Inflammation and infection also induce LD formation in several exposed cell types of the immune system during their activation [175,253,254]. FFAs originate from the neutral core or the phospholipid monolayer and can bind to a variety of cell surface (mainly GPCRs) or intracellular receptors (nuclear receptors) [255]. LD derived PUFAs are precursors for lipid mediators such as eicosanoids, which modulate immune and inflammatory responses [256] and their resolution [257]. LD release eicosanoid precursors from membrane glycerophospholipids by phospholipase A_2_, or from the core by ATGL and HSL mediated lipolysis by [258,259,260]. In addition, cholesterol esters from LDs can contribute precursors upon the action of lysosomal acid lipase [261]. Furthermore, LDs may act as a sink for AA and reduce prolonged and extensive inflammatory responses in immune cells [262,263]. LDs can remove bioactive lipid mediators by inactivation to inert storage lipids, and thereby prevent lipotoxicity. Storage of saturated FAs in neutral TAGs reduces their availability for their inclusion and formation into CEs, thereby reducing inflammatory responses [48]. Similar alterations in neutral TAG levels occur in yeast during aging and apoptosis and provide a potential mechanism to buffer CE-induced stress [81].

Free cholesterol can form cytotoxic cholesterol crystals at high concentrations, which then generate inflammatory responses [264]. The formation and storage of cholesterol esters in LDs protects from cytotoxicity and inflammation [265]. In contrast, during atherosclerotic progression, macrophage LDs accumulate excess cholesterol esters and transform into foam cells, causing inflammation at the arterial walls. Signaling proteins that mediate inflammatory responses are present on LDs of leukocytes [253,266]. Cyclooxygenase-2 is present on LDs and can initiate the production of inflammatory eicosanoid mediators such as prostaglandins and leukotrienes [267,268]. LDs are also a reservoir for lipids involved in intracellular signaling cascades. Members of the PPAR nuclear receptor family are lipid ligand activated transcription factors and play a crucial role in lipotoxicity (see Section 3.1). PPARs control the expression of genes involved in intermediary metabolism, cell differentiation and proliferation as well as inflammation [269]. A fine balance between lipolysis and lipogenesis is required to avoid the accumulation of lipid products that foster IR and inflammation [270,271]. In addition, ATGL activity increases PPAR-α mediated transcription by positive regulation of the deacetylase SIRT1 [272]. SIRT1 can inactivate the transcription factor NF-kappaB and as such, reduce inflammation [273]. The action of ATLG on LDs may initiate additional downstream signaling to SIRT1that includes, FOXO1, PPARs and the transcriptional coactivator PGC1alpha for the coordination of inflammatory responses [274]. It is of special interest that PLIN5 not only regulates lipase activity at LDs [275], and interacts with mitochondria [108], but also binds to SIRT1/PGC1alpha in the nucleus as already discussed in Section 3.2.2 [109]. Among bioactive lipid mediators, omega-6 PUFA derived eicosanoids are important for the initiation and perpetuation of inflammation, although some may even possess anti-inflammatory properties [256]. AA and other omega-6 PUFA are substrates for various enzyme systems including LOX, COX and CYP450. The same enzymes use omega-3 PUFA, such as EPA or DHA for the production of specialized pro-resolving mediators (SPMs), important for the termination and resolution of inflammatory processes [257]. The balance between eicosanoids and SPMs is essential for tissue homeostasis and disturbed in chronic inflammatory diseases [276]. The availability of particular PUFAs and specific lipases in and at LDs, which liberate the substrates for enzymes is crucial for inflammatory processes, especially in cells of the immune system [256,259,277]. LDs may directly release signaling molecules, such as FAs, or precursor for the synthesis of other bioactive lipid mediators, including eicosanoids, isoprostanes, retinoic acid, endocannabinoids, and ceramides. On the other side, the transient accumulation of these lipid mediators in LDs may be important for balanced inflammatory reactions and their resolution [8]. The storage of isoprostanes in LDs could contribute to fine-tuning of the redox state in a response to elevated oxidative stress and prevent protein modifications. The close interaction with organelles that produce ROS, such as mitochondria may facilitate the transfer of oxidized lipid products. The non-enzymatic synthesis of isoprostanoids, inhibits growth and contributes to the induction of LD TAG accumulation in the diatom *Phaeodactylum tricornutum* in response to oxidative stress [9]. The role of LDs as possible storage sites of non-enzymatically synthesized lipid mediators needs further clarification. LDs are clearly at the central hub for controlling inflammatory processes from initiation to termination.

### 5.6. The Role of LDs in Viral, Bacterial and Protozoan Infections

LDs not only contribute to ageing and neurodegenerative diseases, but also in the process of viral infections. Some viruses, such as the hepatitis C virus (HCV) use LDs as a source for the production of infectious virus particles. Acute HCV infection can transform into a persistent infection, and chronic hepatitis may progress to hepatic cirrhosis that can result in hepatocellular carcinoma [278]. For viral replication, free viral core protein is transported to LDs [279]. Steatosis and the accumulation of intracellular LDs is a major complication associated with HCV infection [279].

Synthesis of the 191 amino acid HCV core protein initiates at the ER and involves a C-terminal signal peptide. After cleavage, the core protein transfers to LDs [280] in a mechanism that includes DGAT1. In addition, the prevention of TAG turnover increases the LD number and thus promotes viral assembly [280]. Also the nonstructural HCV protein 5A (NS5A) utilizes LDs as a vehicle. NS5A binds to CD2-associated protein, and the complex then translocates to LDs.

Besides HCV, the life cycle of rotaviruses (RVs) depends on LDs. Infections with RV play important roles in acute gastroenteritis among infants and children [281]. Infections depend on two nonstructural proteins, NSP2 and NSP5. Both proteins are crucial for the formation of viroplasms, the synthesis of dsRNA and virus replication [282]. In detail, viroplasms do interact with LDs via structural features [283]. Especially an amphipatic alpha helix (see Section 3.2.3) in NSP5 may be responsible for its translocation to the LD surface [118]. During RV infection, the lipidome of infected cells significantly changes and levels of CEs, free FAs, phosphatidic acid and sphingomyelins increase. It is interesting to mention, that during RV infection the lipid composition of LD itself remains unaffected [284]. Other classes of viruses utilize LDs to ensure their replication. Dengue virus (DENV), Zikavirus (ZIKV) as well as West Nile virus (WNV) all depend on the LD-associated acyltransferase ancient ubiquitous protein 1 (AUP1) [285]. TAG levels decrease, whereas CE levels increase in ZIKV-and DENV-infected cells. Flavivirus exploits the connection between apoptosis and LDs during late phases of infection. These viruses increase phosphatidylinositol levels to block the induction of apoptosis [286]. During all infections, a defined lipid microenvironment is established inside the cell. This ensures the transport, replication and assembly of core elements needed for pathogen maturation. This is also true for other host pathogen infections. During infections with *Clamydia* as well as the protozoan *Toxoplasma* changes in DGAT catalytic activity have been observed associated with an increased amount of TAGs [252].

All these examples exemplified above strongly suggest that LDs have a central role in a multitude of disease states due its influence on lipid metabolism and its role as a storage site for (harmful) proteins as well as lipids.
